# The additive value of patch testing non‐commercial test substances and patients' own products in a clinic of occupational dermatology

**DOI:** 10.1111/cod.14191

**Published:** 2022-08-03

**Authors:** Kristiina Aalto‐Korte, Maria Pesonen

**Affiliations:** ^1^ Occupational Health Unit Finnish Institute of Occupational Health (FIOH) Helsinki Finland

**Keywords:** 1,3‐benzenedimethanamine, *N*‐(2‐phenylethyl) derivatives (BDMA‐D), allergic contact dermatitis, coco‐amphopropionate, hydrogenated formaldehydebenzenamine polymer (FBAP), in‐house test substances, laboratory technicians, pharmaceutical industry, plants, PVC gloves, synthetic resin systems

## Abstract

**Background:**

Commercial patch test substances do not cover all occupational contact allergens. Workplace materials and in‐house test substances are tested to complement the investigation of occupational skin disease (OSD).

**Objectives:**

To quantify the additional value of testing workplace materials and non‐commercial in‐house test substances in the diagnosis of OSD.

**Materials and Methods:**

Patients files of 544 patients patch tested at the Finnish Institute of Occupational Health in 2015–2019 were reviewed for occupation, diagnoses and patch test results.

**Results:**

OSD was diagnosed in 353 (64.9%) of the patients. A total of 206 (37.9%) patients had occupational allergic contact dermatitis (OACD). In 19 (3.5%) patients, the only clues to the diagnoses of OACD were positive reactions to workplace materials, and in 20 (3.7%) patients, the diagnosis of OACD was based on commercially unavailable test substances. In 167 OACD cases diagnosed by commercial test substances, additional causes were found in 17 by testing patients' own and non‐commercial test substances. In 43 (7.9%) cases, positive reactions to workplace materials reinforced diagnoses based on commercial test substances. The overall additive value of testing own products was 16.7% (91 cases).

**Conclusion:**

We would have missed 39 (18.9%) of our 206 OACD cases if we had solely used commercial test substances.

## Graphical abstract

At the Finnish institute of Occupational Health in 2015–2019, we would have missed 39 (18.9%) of our 206 cases of OACD if we had solely used commercial test substances.

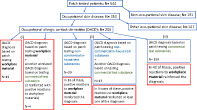

## 1 INTRODUCTION

The investigation of a patient with a suspicion of occupational allergic contact dermatitis (OACD) comprises screening with a baseline patch test series and aimed testing with other patch test series that are chosen according to possible exposures in the job. Products from the workplace are also often tested. In an Australian study, the additive value of patients' own products was 5.2%: in 1.3% of the investigated 1523 cases, a positive reaction to patients' own samples was the only clue for detecting the responsible allergen, and in 3.9% of the patients, testing with their own samples reinforced their reactions to commercial allergens.^1^

At the Finnish Institute of Occupational Health (FIOH), in addition to regular testing with patients' own products, we also prepare in‐house test substances of chemicals that are not available as commercial allergens and include them in our routine test series.

The aim of this study was to quantify the additional value of testing the non‐commercial test substances and patients' own products in the diagnosis of occupational skin disease (OSD) and more closely OACD.

## 2 MATERIALS AND METHODS

FIOH has a clinic of occupational dermatology. All our patients have a suspected OSD. We perform patch tests using Finn Chambers (SmartPractice), in accordance with the European Society of Contact Dermatitis guidelines.^2^ We read the tests two to three times: on D2–D3–D4, D2–D3–D6 or D2–D5, depending on the day of application (Monday, Tuesday or Wednesday). All patients are asked to bring samples of products they handle at work and at home that are possible causes of their dermatitis. We test all the patients with the baseline series, a varying number of special series which are individually chosen based on the patient's occupation and known exposures, and a selection of the samples of own products. After patch tests, exposure to positive allergens is assessed in co‐operation with a dermatologist and a chemist. This assessment often includes time‐consuming inquiries to the manufacturers if a safety data sheet does not provide enough information. We also perform chemical analyses when we fail to show exposure by other means.

A diagnosis of an OACD requires shown exposure to the patch‐test‐positive allergen at the time of the dermatitis investigated, and the mode of exposure must be consistent with the location of the dermatitis.

If a patch‐test‐positive workplace product does not contain any chemical that the patient is allergic to, we try to get the components of the product for a new patch test. If we manage to identify the culprit chemical by the second patch test, we often add this compound to a suitable routine patch test series and start to screen it in patients with similar exposure.

If we cannot identify the causative allergen in a patch‐test‐positive workplace product, we routinely perform patch tests in five control patients before a diagnosis of OACD. When the culprit allergen remains unidentified, the diagnosis is less solid than in cases that are solved to the allergen level. Most new allergens discovered at FIOH have been reported, and in these cases, we usually perform at least 20 tests in control patients.

The study material comprises all cases patch tested with the baseline series in a period of 5 years 2015–2019. We collected data on occupation and diagnosis from the patient files. Only patients diagnosed with OACD were further analysed. They were divided in three groups:

- OACD patients diagnosed by patch testing their own materials.
- OACD patients diagnosed with in‐house test substances that were commercially unavailable at the time of the investigations at the FIOH.
- OACD patient diagnosed with patch test substances that were commercially available at the time of the investigations at the FIOH and not included in Groups i and ii.

We also prepare in‐house test substances (e.g., *m*‐xylylenediamine)^3^ when we want to use higher concentrations than those of commercial test preparations. In the present study, we classified these allergens as ‘commercially available’ test substances.

Some patients had several unrelated causes for their OACD, thus combinations of diagnoses based on commercial test substances and in‐house test substances/own samples were possible. Cross‐reacting allergens were not considered unrelated.

We also noted cases whose positive reactions to workplace materials reinforced OACD diagnoses when they contained an allergen the patient was allergic to. Reinforcements occurred in Groups ii and iii, that is, they fell on positive reactions to both commercial and non‐commercial patch test substances.

## 3 RESULTS

**FIGURE 1:**
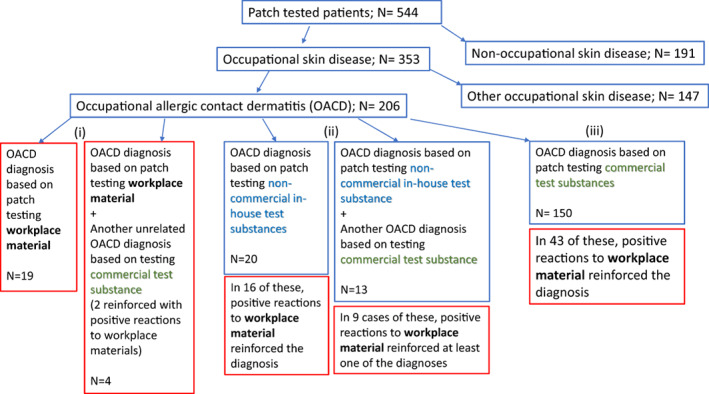
Diagnoses of the 544 patients and subdivision of cases of occupational allergic contact dermatitis into Groups i, ii and iii. One patient may have more than one diagnosis

During the 5‐year period 2015–2019, we patch tested 544 patients (Figure 1). A total of 353 (65%) of them were diagnosed with an OSD. A total of 206 cases (38%) had OACD, 125 (23%) had occupational irritant contact dermatitis and 38 (7%) had occupational contact urticaria or protein contact dermatitis. Multiple diagnoses were possible. Of the total, 541 (99.4%) patients were tested with some materials from the workplace; 92 (16.9%) patients had positive reactions to at least one of their own materials. In one of these patients, the positive reaction was not occupationally relevant (cyanoacrylate‐based eyelash extension glue for personal use).

### 3.1 Patients diagnosed with OACD by patch testing their own products from the workplace (Group i)

In Table 1, we present 23 patients (4.2% of all patch‐tested patients; 11.2% of OACD patients) who had an OACD diagnosis based on positive reactions to materials used at work. These contact allergies were of current occupational relevance, not related to past exposure or symptoms. In 19 of these 23 patients, the workplace material was the only clue that led to the diagnosis of OACD (Figure 1). The remaining four patients had another unrelated cause for their OACD that was diagnosed by commercial patch test substance(s). In two of these four patients, a positive patch test reaction to a workplace material containing the commercial allergen reinforced the diagnosis of OACD.

**TABLE 1:** Group i: Cases of occupational allergic contact dermatitis (OACD) diagnosed by patch testing with workplace materials

	Occupation	Patch‐test‐positive workplace material; positive test methods/concentrations	Other unrelated causes of OACD (corresponding patch‐test‐positive work material)
1	Laundry worker	GT‐Stretch PVC glove; ultrasonic extract	
2	Assistant nurse	Klinion and Saila PVC gloves; as such and ultrasonic extract	
3	Assistant nurse	Klinion and another unknown brand of PVC gloves; as such and ultrasonic extract	
4	Nurse	Klinion PVC glove; as such and ultrasonic extract to acetone (++ reactions); ingredients negative	
5	Chef	Avalon PVC glove; as such	
6	Cleaner	SemperCare PVC glove; as such and ultrasonic extract to acetone	
7	Cleaner	Klinion PVC glove; as such and ultrasonic extracts to acetone and ethanol	
8	Physician	Sempermed Syntegra accelerator‐free rubber gloves (in chemical analysis no thiurams or dihiocarbamates were detected)	
9	Welder	Work trousers; as such and ultrasonic extracts to acetone and ethanol	
10	Kitchen worker	Work blouse; as such and ultrasonic extract	
11	Spray painter	Inerta 165‐01 hardener for epoxy paint 1%–0.32% pet.	
12	Laboratory technician	Diethyl pyrocarbonate 0.2% pet.	
13	Laboratory worker	5‐Chloro‐1‐methyl‐4‐nitroimidazole 10%–0.0032%	
14	Mechanics in the pharmaceutical industry	*N*‐(4)‐(2‐chloropropionyl)phenyl) acetamide 0.2%–0.0002% pet.	
15	Worker in the pharmaceutical industry	*N*‐(4)‐(2‐chloropropionyl)phenyl) acetamide 2%–0.00064% pet.	
16	Gardener	Own chrysanthemums; ultrasonic extract to acetone	
17	Plant keeper	Spathiphyllum^4^; as such	
18	Plant keeper	Spathiphyllum^4^; as such and ultrasonic extract to ethanol	
19	Lash technician	3 lash extension glues 10% pet.	
20	Assembler of electric machines	‘White spot paper’ used in coils; ultrasonic extract to ethanol	Tetramethylthiuram monosulfide
21	Glazier	Sika Aktivator 1 3.2%–0.1% pet: 3‐mercaptopropyl trimethoxysilane^5^ component positive 0.5%–0.016% pet.	2‐Hydroxyethyl methacrylate (own methacrylate‐based resin)
22	Florist	Eucalyptus; as such and ultrasonic extract to ethanol	Tulipalin A (Tulip extract)
23	Welder	Glove (leather); as such and ultrasonic extracts to ethanol and acetone	Cocamide diethanolamide

*Note*: When there is no culprit allergen mentioned, it remained unidentified.

Nine cases were due to contact allergy to protective gloves, mostly PVC gloves, in which the causative allergen remained unknown.

### 3.2 Patients diagnosed with in‐house test substances of commercially unavailable allergens

In Table 2, we present 33 patients who tested positive to in‐house test substances, which lead to the diagnosis of OACD (20 cases; Nos. 1–20) or revealed additional causes for their OACD (13 cases; Nos. 21–33). In the latter group, three patients (Nos. 21–23) had occupational contact allergies to commercial test substances of allergens that were totally unrelated to the allergens of the in‐house test substances. The remaining 10 patients (Nos. 24–33) reacted to in‐house test substances of epoxy hardeners, but they also reacted to other epoxy compounds that were available as commercial test substances. In the whole group of 33 patients, as many as 25 had positive reactions to workplace materials that contained the chemical of the in‐house test substance and thus reinforced the diagnosis of OACD.

**TABLE 2:** Group ii: Cases of occupational allergic contact dermatitis diagnosed by patch testing in‐house test substances of allergens that were not commercially available at the time of the investigation

No.	Occupation	Non‐commercial in‐house test substance	Corresponding patch‐test‐positive workplace material	Other causes of OACD
1	Fast food worker	Coco‐amphopropionate^6^		
2	Fast food worker	Coco‐amphopropionate^6^	Sensisept H34	
3	Cartridge product worker	Coco‐amphopropionate^6^		
4	Fast food worker	Coco‐amphopropionate^6^	Sensisept H34	
5	Fast food worker	Coco‐amphopropionate^6^	Sensisept H34	
6	Fast food worker	Coco‐amphopropionate^6^		
7	Fast food worker	Coco‐amphopropionate^6^	Sensisept H34	
8	Fast food worker	Coco‐amphopropionate^6^	Sensisept H34	
9	Kitchen worker	Coco‐amphopropionate^6^	Sensisept H34	
10	Kitchen worker	Coco‐amphopropionate^6^	Sensisept H34	
11	CNC machinist	Capryl diethanolamine^7^ 2% pet.	Cutting fluid	
12	Car painter	Viapal polyester putty resin 5% pet.	Polyester putty resin	
13	Worker in a paint factory	HDI‐oligomers^8^ 5% pet.	Hardener for a polyurethane paint	
14	Worker in a paint factory	HDI‐oligomers^8^ 5% pet.	Hardener for a polyurethane paint	
15	Tile setter	Solvent Orange 60^9^ 0.1% pet.	Glove (textile part)	
16	Pipe reliner	FBAP^10^ 1% pet.	Epoxy hardener	
17	Painter	BDMA‐D^11^ 0.2% pet.	Epoxy hardener	
18	Painter	BDMA‐D^11^ 0.2% pet.	Epoxy hardener	
19	Painter	BDMA‐D^11^ 0.2% pet.		
20	Floor layer	BDMA‐D^11^ 0.2% pet.	Epoxy hardener	
				Other unrelated causes of OACD
21	Fast food worker	Coco‐amphopropionate^6^		Thiurams, MOR (nitrile glove positive)
22	Machinist	Coco‐amphopropionate^6^	(Sensisept H34 ?+)	IPBC in a cutting fluid
23	Hairdresser	Ethyl hexyl glycerine 2% pet.		Hydroperoxides of linalool
		Epoxy hardeners		Other epoxy‐related causes of OACD
24	Boat builder	Tetraethylenepentamine 1% pet.	Epoxy hardener	IPDA, TETA
25	Pipe reliner	TMD 0.32% pet.	Epoxy hardener	Bisphenol F epoxy resin, MXDA, BDDGE
26	Pipe reliner	TMD 0.32% pet.	Epoxy hardener	Bisphenol A/F epoxy resins, TETA, BDDGE
27	Welder/sheet metal worker	tris‐DMP^a^ 0.32% pet.	(tris‐DMP‐containing hardener ?+)	Bisphenol A epoxy resin
28	Wood product worker	FBAP^10^ 1% pet.	Epoxy hardener	Bisphenol A epoxy resin
29	Plumber	FBAP^10^ 1% pet.		MXDA
30	Floor layer	FBAP^10^ 1% pet.	2 epoxy hardeners	MXDA, IPDA
31	Pipe reliner	FBAP^10^ 1% pet.	Epoxy hardener	Bisphenol A/F epoxy resins, MXDA
32	Worker in a paint factory	BDMA‐D^11^ 0.2% pet.	Epoxy hardener	MXDA
33	Painter	BDMA‐D^11^ 0.2% pet.	Epoxy hardener	Bisphenol A epoxy resin

*Note*: For further details, please read the references. Coco‐amphopropionate was tested with a dilution series of 10%, 3.2% and 1% in pet.

Abbreviations: BDDGE, 1,4‐butanediol diglycidylether; BDMA‐D, 1,3‐benzenedimethanamine, *N*‐(2‐phenylethyl) derivatives; FBAP, hydrogenated formaldehydebenzenamine polymer; HDI, hexamethylene diisocyanate; IPBC, iodopropynyl butylcarbamate; IPDA, isophorone diamine; MOR, 2‐(4‐morpholinylmercapto)benzothiazole; MXDA, *m*‐xylylenediamine; NT, not tested; OACD, occupational allergic contact dermatitis; TETA, triethylenetetramine; TMD, trimethylhexamethylenediamine; tris‐DMP, 2,4,6‐tris‐dimethylaminomethylphenol.

^a^ The case was investigated in 2015, when tris‐DMP was not yet available from Chemotechnique.

Twelve cases of coco‐amphopropionate contact allergy were diagnosed during the 5‐year study period. They were all caused by Sensisept H34 disinfectant hand cleanser that has been extensively used especially in fast food restaurants.^6^

### 3.3 Patients diagnosed with commercially available allergens and having positive reactions to own materials that reinforced the diagnosis of OACD (Group iii)

In further 150 cases, the diagnosis of OACD was based on positive reactions to commercially available test substances. In Table 3, we present 43 of them who tested positive to workplace materials that reinforced the diagnosis of OACD. We did not include patients already presented in Tables 1 and 2 (Groups i and ii). The largest group in Table 3 was 23 cases caused by epoxy compounds. Five cases were caused by thiurams, four by acrylic compounds, three by phenol formaldehyde resins and three by isocyanates/4,4′‐diaminodiphenylmethane.

**TABLE 3:** Patients with occupational allergic contact dermatitis diagnosed with commercially available allergens displaying concomitant positive reactions to corresponding own products that contained the patch test‐positive allergen(s)

No.	Occupation	Patch test‐positive commercially available allergen(s)	Own products containing the same allergen(s)
1	Concrete worker	Tetraethylthiuram disulphide (TETD)	Rubber glove^a^
2	Process worker	Thiurams	Rubber glove^a^
3	Chef	Thiurams	Nitrile glove^a^
4	Nurse	Thiurams/dithiocarbamates	Rubber gloves^a^
5	Nurse	Tetraethylthiuram disulphide (TETD)	Ultrasonic extract of rubber gloves^a^
6	Farmer	Glutaraldehyde	Disinfectant
7	Farm relief worker	Colophony	Saw dust used as animal bedding
8	Cosmetic shop assistant	Hydroperoxides of limonene and linalool	‘Gelee Auto Bronzante’ Self‐tanning cosmetic product
9	Glass fibre worker	Hexanediol diacrylate (HDDA)	Resin
10	Assembler	2‐hydroxyethyl methacrylate (2‐HEMA)	Anaerobic glue
11	Hairdresser	Ethyl cyanoacrylate	Lash extension glue
12	Glazier	2‐hydroxyethyl methacrylate (2‐HEMA)	Resin
13	Printer	Methylchloro/methylisothiazolinone	Additive for fountain water
14	Florist	Tulipalin A	Tulip stem
15	Engineer	Phenol formaldehyde resin (PFR2)	2 resins
16	Plywood worker	Phenol formaldehyde resin (PFR2)	Resin
17	Plywood worker	Phenol formaldehyde resin (PFR2)	Resin
18	Floor layer	MDA, MDI, PMDI	MDI‐containing hardener
19	Pipe reliner	MDI, PMDI	Hardener
20	Spraypainter	MDA	Hardener
21	Assembler	Bisphenol F epoxy resin, BDDGE	Resins
22	Pipe reliner	Bisphenol A epoxy resin, MXDA	Resin and hardener
23	Bricklayer	IPDA	Hardener
24	Pipe reliner	Bisphenol A epoxy resin	Resin
25	Plumber	Bisphenol A epoxy resin	Resins
26	Insulation worker	Bisphenol A/F epoxy resins, MXDA	Resin and hardener
27	Painter	Bisphenol A/F epoxy resins	Resin
28	Plumber	Bisphenol A/F epoxy resins, MXDA	Resin and hardener
29	Pipe reliner	Bisphenol A epoxy resin	Resin
30	Foreman	Bisphenol A epoxy resin	Resin
31	Pipe reliner	Bisphenol A/F epoxy resins, MXDA	Resin and hardener
32	Construction worker	Bisphenol A epoxy resin, MXDA	Resin and hardener
33	Pipe reliner	Bisphenol A/F epoxy resins	Resins
34	Pipe reliner	MXDA; BDDGE	Resin and hardener
35	Tile setter	Bisphenol A/F epoxy resins; IPDA	Resin and hardener
36	Pipe reliner	Bisphenol A/F epoxy resins; MXDA	Resin and hardener
37	Lamination worker	Bisphenol A epoxy resin, BDDGE	Resin
38	Plumber	Bisphenol A epoxy resin; MXDA, IPDA	Resin and hardeners
39	Construction worker	MXDA, IPDA	Hardener
40	Manufacturer of prefabricated units	Bisphenol A/F epoxy resin	Resins
41	Boat builder	MXDA, triethylenetetramine	Hardeners
42	Assembler of electric machines	Bisphenol A epoxy resin	Insulation tape
43	Pipe reliner	Several reactive diluents/epoxy resins^b^	Resin

Abbreviations: BDDGE, 1,4‐butanediol diglycidylether; IPDA, isophorone diamine; MDA, 4,4′‐diaminodiphenylmethane; MDI, diphenylmethane‐4,4′‐diisocyanate; MXDA, *m*‐xylylenediamine; PMDI, polymeric diphenylmethane‐4,4′‐diisocyanate.

^a^ We did not analyse the gloves, but assumed that they contained dithiocarbamates that cross‐react with thiurams.

^b^ The patient had positive reactions to a large number of reactive diluents and cross‐reacting epoxy resins, but the exact compound in the patch test ‐positive resin could not be determined; bisphenol A epoxy resin was possibly falsely negative at FIOH ‐ it had been positive in a local hospital. The patient was exposed to bisphenol A epoxy resin.

## 4 DISCUSSION

Over the decades, we have developed special patch test series for workplace materials such as epoxy chemicals, isocyanates, phenol formaldehyde resins and metalworking fluids by adding new in‐house test substances. These additions have been allergens discovered in our clinical investigations, suggested by the literature, or based on our own research on chemical exposure in workplaces. In this study, we wanted to assess the additive value of patch testing not only workplace materials but also our numerous in‐house test substances that have not been commercially available.

In the previous literature, there is a report from an Australian clinic of occupational dermatology by Slodownik et al.^1^ that assessed the additive value of patch testing patients' own products in 1532 consecutively tested patients. Although their material was much larger than ours, they reported no more than 101 patients who reacted positively to their own materials while the corresponding figure was 91 in the present material. The Australians did not report the numbers of OACD diagnoses or patients tested with their own materials. These two figures were high in our study (38% and 99%, respectively). In the Australian report, in 20 patients (1.3% of the total) a positive reaction to their own materials was the only clue to the diagnosis (‘definite additive value’) and for 59 (3.9%) cases, testing with their own products reinforced the diagnosis based on reactions to commercial allergens leading to 5.2% (*n* = 79) ‘overall additive value’ of patch testing with patients' own products. The corresponding absolute figures in our material were quite similar (19 [3.5%], 72 [13.2%] and 91 [16.7%], respectively), but the proportions were around triple‐fold due to the almost three‐fold total number of investigated patients in the Australian study.

A recent German study assessed the value of patch testing with workplace materials in occupational dermatitis patients.^12^ In a material of 654 patients with suspected occupational dermatitis, they found 23 positive patch test reactions to patients' own materials that were not accompanied by corresponding reactions to standardized patch test preparations. The number is of the same order as in the present and the Australian studies, provided that each reaction belonged to a separate patient. It was not stated if multiple reactions per patient occurred, and only 12 reactions were regarded as currently occupationally relevant. The number of positive reactions to patients' own materials was 154, but, again, the number of corresponding patients might have been lower. At FIOH, multiple positive reactions to workplace materials per patient were common, and thus the number of individual reactions was much higher than 91. The Germans diagnosed OACD in 17.3% of the patients, a figure much lower than ours.

In the German study,^12^ the largest patch‐test‐positive product types were metalworking fluids, leave‐on products, hand or skin disinfectants, and gloves. In the present study, various resins and their hardeners were by far the most important workplace materials in Groups ii and iii, while PVC gloves were prominent in Group i. In line with our results, the Australian report comprised many cases of contact allergy to resins and adhesives, and patch‐test positive rubber gloves were also quite numerous.^1^

The additive value probably depends on the patient material (proportion of OACD patients) and the extent of patch testing of patients' own materials. It also depends on how well the culprit products are identified and selected for patch testing, a task that can be very challenging, if the number of products is large. Moreover, not all products are ideal for patch testing, and they may require special techniques such as ultrasonic extracts. Positive reactions to workplace materials are more common in strongly sensitized patients.

The occupational dermatology unit of the FIOH is a special clinic with expertise on patch testing patients' workplace materials. That is why we receive patients who have handled sensitizing chemical products at work. Our patient material is very selected and might differ from that of other centres.

Laboratory technicians and workers in the pharmaceutical industry are often exposed to chemicals that are not included in commercial patch test series. Commercial plant allergen preparations are also scarce, and own plants need to be tested in florists and gardeners.

We consider protective gloves as problematic products: Although we have identified several allergens in PVC gloves^13^, ^14^, ^15^ over the years, the present material still comprises seven cases due to PVC gloves in which the culprit allergen remained unknown. The same applies for textiles. In Finland, allergic contact dermatitis caused by allergens in the current commercial textile colour and finish series is rare. Only occasionally we have managed to reveal a culprit allergen in textiles.^9^

Diagnosing OACD due to synthetic resins and their hardeners has been relatively straightforward: manufactures have usually co‐operated by sending components of their products for patch testing. Two new epoxy hardeners, 1,3‐benzenedimethanamine, *N*‐(2‐phenylethyl) derivatives^11^ and hydrogenated formaldehydebenzenamine polymer,^10^ identified at FIOH have proved important as the present material comprises 11 cases caused by them.

## 5 CONCLUSION

We would have missed 39 (18.9%) of the 206 OACD diagnoses if we had confined our patch testing to commercial patch test substances. In 16 (7.8%) other cases, testing with workplace materials and in‐house test substances yielded additional causes for OACD. In further 43 (20.9%) cases, positive reactions to workplace materials reinforced the diagnosis of OACD made by testing commercial test substances. Thus, in a total of 98 (47.6%) cases of OACD, the diagnostic workup was aided by testing workplace materials and non‐commercial in‐house test substances.

## AUTHOR CONTRIBUTIONS

**Kristiina Aalto‐Korte:** Conceptualization; investigation; writing – original draft; data curation; visualization; methodology. **Maria Pesonen:** Writing – review and editing.

## CONFLICT OF INTEREST

The authors declare no conflict of interest.

## Data Availability

The data that support the findings of this study are available from the corresponding author upon reasonable request.
